# Building a protein name dictionary from full text: a machine learning term extraction approach

**DOI:** 10.1186/1471-2105-6-88

**Published:** 2005-04-07

**Authors:** Lei Shi, Fabien Campagne

**Affiliations:** 1 Institute for Computational Biomedicine and Dept. of Physiology and Biophysics, Weill Cornell Medical College; 1300 York Ave; New York, NY 10021, USA

## Abstract

**Background:**

The majority of information in the biological literature resides in full text articles, instead of abstracts. Yet, abstracts remain the focus of many publicly available literature data mining tools. Most literature mining tools rely on pre-existing lexicons of biological names, often extracted from curated gene or protein databases. This is a limitation, because such databases have low coverage of the many name variants which are used to refer to biological entities in the literature.

**Results:**

We present an approach to recognize named entities in full text. The approach collects high frequency terms in an article, and uses support vector machines (SVM) to identify biological entity names. It is also computationally efficient and robust to noise commonly found in full text material. We use the method to create a protein name dictionary from a set of 80,528 full text articles. Only 8.3% of the names in this dictionary match SwissProt description lines. We assess the quality of the dictionary by studying its protein name recognition performance in full text.

**Conclusion:**

This dictionary term lookup method compares favourably to other published methods, supporting the significance of our direct extraction approach. The method is strong in recognizing name variants not found in SwissProt.

## Background

Knowledge discovery and data mining in the biological literature have been attracting more and more interest [1,2]. Automated text mining can facilitate the efforts of both biological database curators [2], and of biologists who consult the literature to acquire novel information both within and outside of their immediate expertise. Text mining applications come in various styles. Some rely on statistical methods to detect unusually strong co-occurrences between genes or gene products (e.g., PubGene [3] and as described by Wilkinson et al. [4]). Other applications aim to extract precise information from the text, for instance protein mutations [5] or interactions [6,7]. A new promising type of application, pioneered by Textpresso [8], consists of portals that help end-users locate information more effectively.

Most text mining applications require the ability to identify and classify words, or multi-word terms, that authors use in an article to refer to biological entities (biological entities include, but are not limited to, genes and their products, cell types, and biological processes). This task is called named entity recognition and has been well studied in computer science for problems such as recognizing names of places, people and organizations in news articles [9]. Efforts to automate the named entity recognition task in the news domain have been very successful, with accuracies of automated methods that compete with accuracies of human efforts (~95% [9]). However, adapting these methods to the biomedical literature has been challenging [10,11], and recently published methods on text from abstracts report accuracies around 75% for protein names [11,12].

Several strategies have been tried to recognize biological entity names in articles. Some methods rely on protein and gene databases to assemble dictionaries of protein names. A key disadvantage of these methods is that they critically depend on the database used as source of names, and will not recognize new protein names before the name is entered in the database. Another problem is that databases may not contain each name variant used to refer to a protein, so that partial or fuzzy matching of the names in the database to the text in the article is needed [13]. Fuzzy matching is difficult to control for and may introduce spurious matches. Another class of methods attempt to recognize protein names using the morphology of the term: whether a term contains mixed case, or includes a prefix or suffix next to a protein name [14]. The main advantage of these methods is that they can detect protein names that authors have just created, if they follow the morphological clues that the method recognizes. The main disadvantage is that authors do not use morphology consistently, and that certain terms of the language that have been used to refer to proteins (e.g., MAD and Eye, [FlybaseIds FBgn0011648 and FBgn0000624]) cannot be recognized based on morphology [10].

Most of these methods were developed for abstracts, because abstracts are readily available for millions of articles (e.g., PubMed). However, it is clear that information content in text from full length articles is much greater than in abstracts, even if the information density (information content divided by document length) is higher in abstracts [15,16]. Specifically, as noted by Horn et al., information about protein mutations is much more likely to be found in full length articles than in abstracts [5]. This is most likely to be true of other types of information as well, such as experimental techniques and protocols, cell types, species names, and interactions. Since full texts are becoming more accessible (with open access efforts such as the Public Library of Science, PubMed Central or BioMed Central), developing methods to extract information from full text is becoming more practical.

Compared to abstracts, however, full length articles are a complex source of text. They usually require pre-processing to convert the document from the HTML or PDF format to plain text streams. The result of the conversion will contain all the sections of the article and separating sections is a hard problem in itself because formatting styles and conventions vary across journals, and change periodically even for a given journal. In addition, journal presentation styles are interleaved with the text of the article and require specialized processing so that the tokenization of the document matches the way the document looks when rendered in a web browser. For instance, removing all HTML tags when converting an HTML document to text is inappropriate, as this may create tokens that do not exist in the article (for instance, when removing all HTML tags, "morphology<P>Browser" could be interpreted as "morphologyBrowser"). Although some measures can be taken to limit these problems, in practice, conversion procedures are difficult to fine tune for each style and journal, and converted full length articles are noisy sources of text. Therefore, methods designed to extract named entities from full text must be robust to types and amounts of noise which are not found in abstracts.

In this article, we present a method that extracts biological named entities directly from full length articles. This method is used to process a corpus of 80,528 full text articles and assemble a catalog of protein name references indexed by an article PubMed identifier (PMID) with high precision. Based on this catalog, we construct a dictionary of 59,990 protein names. We also present a method that uses this dictionary to identify the set of protein names a full text article refers to. We evaluated the performance of this method with a set of full text articles that was not used for the construction of the catalog. The method evaluated on a test set of 14 articles achieves an average precision of 75%. Most other published methods have been trained and tested on abstracts only (e.g., GAPScore, YAGI and NLProt [12,14,17]). While these methods were not developed for full text, they are representative of the state of the art for protein name extraction. We thus chose to compare the results obtained with our approach to the results obtained with NLProt and YAGI, when used on the same text material. We found that the performance of our method compares favorably to the 42% average precision that NLProt achieves on the same test set, at similar levels of recalls. Tested on another set, our approach has better average precision than YAGI, but fails to detect long protein name variants that are often detected by YAGI. These results demonstrate the usefulness of direct name extraction from full text articles.

We have implemented the approaches described here in the Textractor framework .

## Results

### High frequency terms in biological journal articles are enriched in biological entity names

Many biological research journal articles focus on one or a few genes or gene product(s). Typical examples are articles that describe the cloning of a particular gene. Other articles that describe the discovery of a new interaction will often focus on the two gene products involved in the interaction. Thus, the high frequency terms in these articles often refer to a gene/protein, the cell line in which the gene/protein is being studied, or the biological process in which the gene/protein is involved. Setting a threshold on the frequency of terms in an article allow us to define the set of terms that are most frequent in the article. In this study, we used a threshold of 30, and thus we considered "most frequent" a term that occurred at least 30 times in a given article (see Table 1).

**Figure 1:**
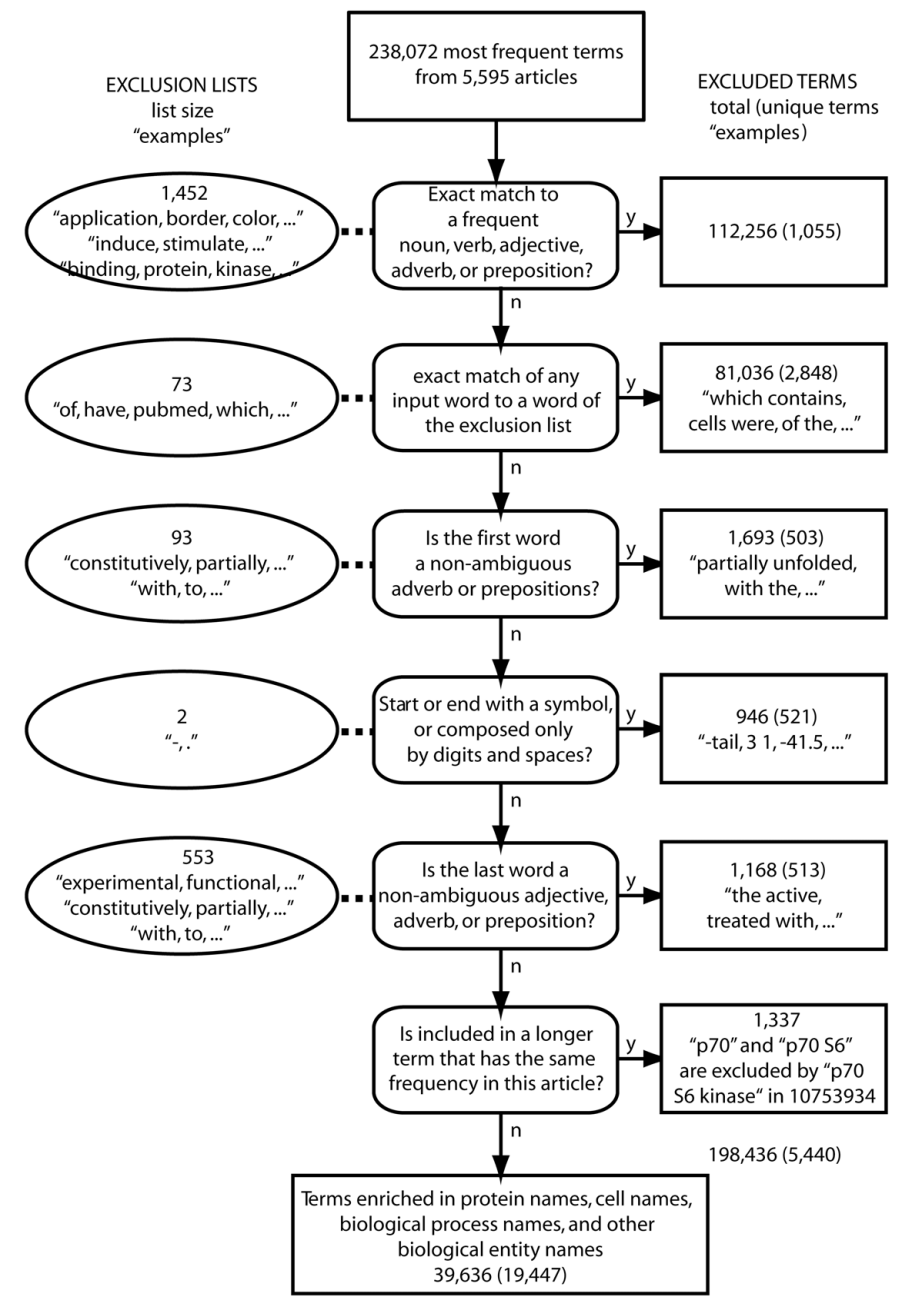
**Filtering process and exclusion lists. **Oval boxes on the left of the figure present the exclusions lists used as input to the filtering process. An exclusion list is connected to the step of the filtering process that uses it. Each step filters out terms that match the exclusion list in sequence based on the rules described in the boxes of the second column. Some steps perform matches by considering the entire term (e.g., the first step on the top), other steps use only specific words in a term. These lists have been built and are being maintained manually. The rectangular boxes on the right show the number of terms that have been excluded at each step, when processing the most frequent terms from JBC2000 (numbers in parentheses indicate counts of unique terms).

Table 1 lists the most frequent terms found in one example article, and how we sort them into two categories. Category (a) includes names of biological entities. Category (b) includes terms that may have a high frequency in an article, but are not biological entity names. Terms in category (b) include common words or combinations of common words (e.g., "the", "1", "figure", "the protein", "binding"). It is possible to build an exhaustive list of terms in (b) because most of them tend to be repeated from article to article. We have created a filter that can remove most terms from category (b) from the list of frequent terms in an article. Figure 1 presents the algorithm and information that this filter uses. An exhaustive list, however, cannot be built for terms in category (a) because, as with protein names, this list is potentially infinite: the rules that authors use to define new names are only bounded by their imagination. We applied a machine learning strategy to differentiate and classify terms that belong to category (a).

### Learning and classifying with the neighboring features

Three support vector machine (SVM) models were constructed to predict if a term occurs in a context that indicates (1) a protein *versus *a cell, (2) a protein *versus *a process (3) a protein *versus *an interaction (See Materials and Methods).

Since each of the most frequent terms occurs at least 30 times in a given article, we can make at least 30 predictions for one term in one article. We expect that some occurrences of a term will occur in contexts that do not clearly indicate if the term is a protein or not. For instance, in a sentence such as "*The exact [stoichiometry of this > multienzyme complex < is unknown but] its molecular mass in insect cells...*", the context included in the window offers little clue to decide if the term refers to a protein or not (The occurrence of the term under inspection is marked by > < and terms considered as features in the window around the occurrence are enclosed in [ ]). In such cases, the SVM score is expected to be small. In comparison, occurrences like "*...is restricted to skeletal muscle and does not affect [expression of the >Glut4< isoform]*.", are very informative since the features "expression" and "isoform" are present together in the local context (note: if "*isoform*" always occurs after "*Glut4*" in that article, in our algorithm, "*Glut4 isoform*" may also be extracted by our system).

We used a simple heuristic expression to combine the classification results of these three models (See Materials and Methods) and created a protein name catalog for all the articles in the JBC2000 dataset.

### Quality of the protein name catalog

**Figure 2:**
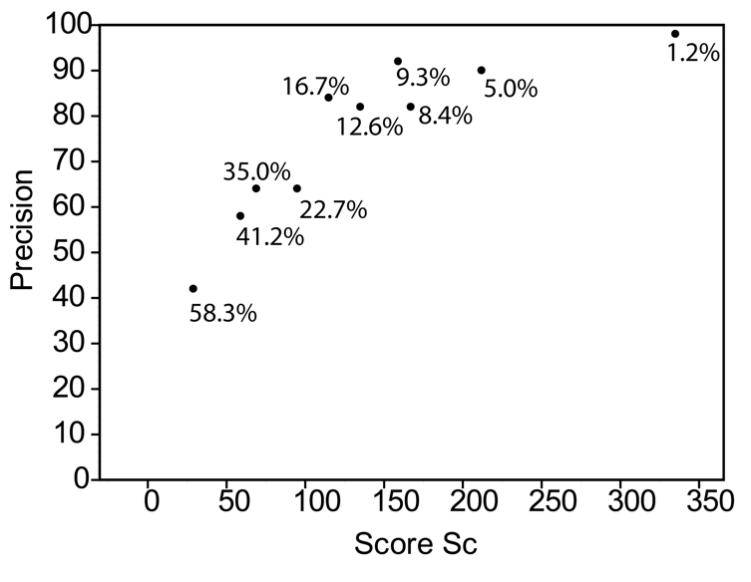
**Relation between the precision of the disambiguation and score values**. Ten score values were chosen randomly. For each of these values, we considered the 50 terms that had scores immediately greater than the value, and evaluated if the term referred to a gene or gene product in the article where the prediction was made. Precision of the prediction was calculated as the number of correct predictions over the total number of predictions (50). The 500 names which were checked represent a random sample of 21,501 names in the catalog. The Figure shows that predictions made with higher scores have a greater probability to be correct. The label shown for each evaluation point indicates the percentage of terms found in the evaluation corpus with a score above the score of this specific point.

For the JBC2000 dataset, we predicted a catalog of 21,501 protein names and ranked them by their combined scores (Sc). The Sc distribution was evenly divided into 10 buckets. One continuous score range was then randomly selected in each of the 10 buckets, so that each range includes 50 protein names. We evaluated the quality of this catalog by manually verifying this random sample of 500 names in the corresponding articles. Figure 2 summarizes this evaluation and provides precision measures. From JBC2000, we estimate that, on average only 3 or 4 protein names will be extracted from a given article. Since this number of names extracted is very low compared to the complete set of names that an average article contains, we have not evaluated the coverage of the prediction. However, because protein names are reused in different articles, if the method misses a name in one article, this name may still find its way into the protein name dictionary through another article (see below). A more interesting evaluation is thus how well would this catalog do in a specific application, such as extracting unique protein names in a full text article. This is the evaluation that we present in the rest of this article.

### Quality of the protein dictionary

**Figure 3:**
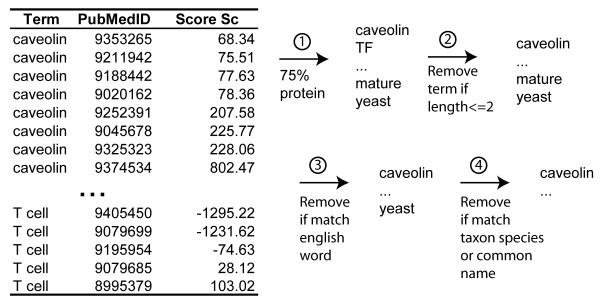
**Heuristics filtering of the protein catalog to produce the protein dictionary. **The dictionary was constructed by considering the classification results of a particular term in different articles. Step 1: we filtered out terms that were predicted to be a protein in less than 75% of the articles where a prediction was made. For example, if term A appears in 4 articles and is classified as a protein name in 3 of them, term A is accepted in the dictionary. This process collected 61,312 terms. Step 2: we removed terms with two characters or less. Step 3: to remove ambiguity with protein names that are also common nouns, we filter the dictionary against the Webster's Revised Unabridged Dictionary (G & C. Merriam Co., 1913, edited by Noah Porter, provided by Patrick Cassidy of MICRA, Inc, and retrieved from ). We estimate that this edition contains about 80 common protein names (e.g., amylase). Step 4: we filter the dictionary against species names from the NCBI taxonomy database [30].

To address this question, we created a protein dictionary from a more comprehensive protein catalog, which is based on 80,528 full text articles (from JBC, EMBO, PNAS) (see Figure 3). We used names in the dictionary to lookup protein names in a set of 14 full length articles from *Nature Cell Biology *(NCB14). The tool used to lookup protein names is provided in Additional File 1.We used the relative recall measure to compare our dictionary-based method to NLProt on NCB14 (See Materials and Methods). Table 2 summarizes these precision and relative recall measurements. These data indicate that the lookup approach that we implemented in Textractor outperforms NLProt on full text articles in precision (75% compared to 42%) at the same level of relative recall (See Materials and Methods for our definition of "relative recall').

During the evaluation on NCB14, we noticed that NLProt made systematic errors on tokens that appeared in the text of these articles because of journal style formats (e.g., mistakes were made for instance on terms such as "NPG" for Nature Publishing Group, or "getObject(name)" which was part of the NPG Javascript header that our pre-processing protocol failed to remove). These terms were mistaken for gene/protein names. To rule out any possible effect due to the selection of the articles in NCB14 (all selected from the same journal), we performed a second evaluation on 15 randomly selected articles from PubMedCentral (published in 2003 and not part of our training set). Data in Table 3 confirm that Textractor outperforms NLProt on full text (see relative F-1 measure).

Table 3 also shows the results of the comparison between Textractor and YAGI [17]. While Textractor outperforms YAGI in term of precision, the recall of YAGI is more than double the recall of Textractor. This is summarized by a relative F1-measure of 51% for YAGI, and 42% for Textractor.

Inspection of the validation data suggests that YAGI is very good at identifying protein names variants that are longer (word length) than the ones that Textractor typically detects (e.g., in article with PMCID 150636, when Textractor identified NS4B as a protein name, YAGI also identified "hepatitis C virus nonstructural protein NS4B"). This point also illustrates a difficulty of comparing protein name extraction methods, since the total set of correct names may include redundancy.

In our evaluation, we have chosen to count as correct names those that refer to proteins by themselves, even if such names are shorter versions of longer names. These shorter versions are not partial names, however, because their occurrence in the text is sufficient to recognize a reference to a specific protein.

Since YAGI and NLProt use similar sources of information, including protein names derived from biological databases, but differ in their machine learning approach, these results also suggest that Conditional Random Fields (see [18], used by YAGI), were able to better capture the distribution of protein names in full text, when learning on abstracts, than the learning approach used by NLProt. To corroborate this conjecture, many errors made by YAGI seem to appear in the reference section of articles, which has a different structure than the rest of a full text article. Since both YAGI and NLProt use a database derived dictionary, it would be interesting to see if the precision of these approaches on full text can be increased when using a dictionary built directly from full text.

### Limited overlap with SwissProt derived names

We matched terms in the dictionary to description lines in SwissProt (release 45), as described for matching terms in the dictionary to full text articles. We found that only 8.3% of the terms in our dictionary are included in SwissProt descriptions. Examples of terms present in the dictionary that do not match SwissProt description lines include: "collagen XI", "Collagen alpha1 I", "TRHRs", "TRIM8 GERP". This result indicates that our method is strong at identifying protein name variants that are not found in SwissProt descriptions. SwissProt is a main source of protein names used for building protein name recognition methods [12,13,17]. Therefore, combining our dictionary with names derived from SwissProt may lead to improved performance for a variety of name recognition methods.

### Implementation and performance

We implemented the methods described in this manuscript in the textractor framework (L. Shi and F. Campagne, unpublished). Briefly, this framework provides support to parse full length articles into sentences; create inverted indices where sentences are indexed by words; store sentences, articles and other information in a database; calculate features from sentences, articles or part of the above; and import results of predictions made with the features into the database for integration with other types of information. The Textractor framework uses JDO [19] for data persistence, MG4J [20] for inverted-index support, and SVMLight [21] for machine learning. The framework is implemented in Java 1.4+. The distribution includes a version of the lookup tool that functions independently of a database and uses MG4J for fast term lookups.  This tool is distributed as Additional File 1. With a dictionary containing about 59,000 names, processing a set of 30 articles (133,675 words total) took about 16 seconds on a Red Hat Linux dual Xeon 3 GHz processor server, while it took NLProt 19 minutes, and YAGI 29 seconds. Similarly, YAGI processed a set of 693 articles in 753 seconds, while Textractor needed 494 seconds (65% of the time required by YAGI).

We distribute the implementation of our method and other relevant information, e.g., the list of regular expressions used to extract the terms for SVM training, under the Gnu General Public License to maximize their use in the biomedical community .

## Discussion

### Information in full text

Compared to full text, abstracts have the advantages of convenient access and uniformity of format. Based on recent studies, however, the information content in abstracts is less than half the information content in full text [15,16]. In addition, most experimentalists would consider full text a more informative source of information than abstracts. Indeed, an abstract usually focuses on the general idea of a biological issue *per se*, but the details, such as how the issue was studied in that article, are very unlikely to be fully described in an abstract. For an experimentalist, the question is more often "how" than "what" and is always about details. Thus, automatic text mining applications that target experimentalists should ideally also be able to extract relevant information from full text articles.

This study addresses an initial step in this direction – to collect and classify several categories of biological entity names from full text that are essential for a detailed understanding of an issue, including how it was studied, from a biological article. These categories include the terms that refer to protein/gene, cell type, biological process, and interactions. Specifically, we compared the recognition of the protein/gene names with other systems and emphasized the strength of our approach in identifying term variants.

### Term variants and disambiguation

Our method draws on several concepts introduced in [22], but differs in the following ways. (1) We use only features derived from the context of the term. This makes our method insensitive to the morphology of the term and allow us to collect terms as diverse as "d2-dopamine receptor", "dopamine D2 receptor", "D2 dopamine receptor", "D2R", "D2DR", "DRD2", "d2s receptor", "d2l receptor" and their plural forms for the same protein in the catalog. (2) We applied term disambiguation to terms extracted from full text without the help of a dictionary of protein names. In contrast, [22] used Genbank as the source of its dictionary and extracted terms by fuzzy-matching the text of the article. (3) We combine information from the multiple sentences in which a term occurs to make a prediction if the term is a protein or not in a given article. Since we disambiguate only terms that appear more than 30 times in an article, information from at least 30 sentences is considered for each term in an article. (4) The precision measures that we report benchmark the ability of our method to precisely identify that a term is a protein in a given article. Performance measures given in [22] measure the performance of a related, but distinct task: assigning a term to one of three classes (protein, mRNA, gene) when it is already known that the term belongs to one of these classes.

### Extraction of long protein names

We have described that our method may fail to detect long protein names (see Results). Such names appear with a lower frequency in articles and are often abbreviated in full text. Approaches to extract acronym definitions from full-text may complement our approach and help extract long protein names (see [23] for a review of such approaches).

### Specific evaluation protocol for full-text

Protein name marking is a task where a program tries to mark the occurrences of all the protein names in a text. In full text, some protein names are repeated very often. If we followed the name marking benchmark standards, we would count correct extractions several times for such repeated terms. While this is not a problem for abstracts, we noticed that this practice would artificially inflate the precision measures on full text.

Instead of benchmarking the performance of gene/protein name tagging in the text, we thus benchmark the performance of extracting the names of gene/proteins mentioned in a given article. We evaluate this by counting errors and success at the level of unique terms. Since this protocol is different from the ones used in other published studies, performance measures that we report cannot be compared to other published measures. Another factor that renders such comparison meaningless is the difference in the input material. We show in Tables 2 and 3 that the performance of a given method varies widely from article to article. Our validation protocol, however, compares the performance of several methods on the same input material, using the same evaluation measures and therefore supports objective comparisons between the methods benchmarked.

### Relative recall helps compare several methods

Obtaining accurate measures of coverage is challenging because coverage requires counting the number of correct protein names in an article. Several factors make obtaining these counts more difficult for full text than for abstracts. (1) Full texts refer to a large number of protein names in all the sections of the paper (in the first test set, NCB14, we recognize on average a total of 58 unique names per article). (2) It is difficult for human annotators to identify all spelling variants of protein names that automated methods may identify. To alleviate the impact of these factors on the evaluation, we presented annotators with names identified by each prediction method. In practice, this procedure guarantees that annotators consider each name variant predicted by one of the methods and determine if the name refers to a protein. The protocol thus helps avoid omissions that can occur when annotators are not familiar with the subject of the article, and directly provides the annotation counts (Ci) required to calculate the relative recall measure (see methods).

### Building the catalog

Our method creates a catalog of protein and gene name references directly from full length noisy article materials. Tanabe and Wilbur recently described a method to assemble a gene/protein lexicon from the text of abstracts [24]. While there is certainly an overlap between protein names used in abstract and in full text, it is not clear what the extent of the overlap is. Thus, our method and the one presented by Tanabe and Wilbur should be complementary. Finally, our study of the quality of the protein catalog in a common application (protein name recognition in full text material), demonstrates the utility of direct name extraction from full text articles.

## Conclusion

Our results show that a pure dictionary-based lookup method can outperform NLProt [12] on full text articles, when using a dictionary built directly from the same type of source material (full length articles). We have presented a method that can build such a focused dictionary from a large corpus (> 80,000) of articles. The method is computationally efficient. We freely distribute the dictionary that we have built to carry out our evaluations and a program that can extract names of proteins from full length articles.

Our method is robust to various sources of noise found in full length articles and achieves a state of the art level of precision on this material. A key element that contributes to the robustness of our method seems to be that we never extract a name from an article as a protein name based only on the morphology of the term, but instead require that the term is predicted several times as a protein name in other articles. This robustness may be the result of considering an array of evidence, found in the sentences from several articles, to determine if a term is likely to refer to a gene or gene product, and reusing this knowledge again and again. In its current state, an outstanding limitation of our approach is its inability to deal with certain types of term ambiguity (i.e., the same term referring to a protein ("TnT"/Troponin T or "TnT"/Translation and Transduction kit). This is an area for improvement that will require further research.

## Methods

### Document pre-processing and extraction of terms

We represent all documents with the Unicode standard [25]. We define a *word *as a contiguous sequence of letter, digits, dashes and dot characters (we use the classification of Unicode characters established for the Java language and implemented in the java.lang.Character class of this language, so that letters include special characters such as α or β). We split documents into sentences of at least *m *characters, with heuristics that consider the types or identity of characters in a window of 6 characters around potential sentence terminators (characters .;?!). Parameter *m *was set to 40 in this experiment, a value that rejects sentence splits if they would create a sentence shorter than about a quarter of a column of this article. Full details of the sentence splitting procedure are given in the source code in the supplementary material (see class SentenceSplitterIterator).

We define a *term *as a word or a contiguous sequence of words, which appears in an article, but does not span sentence boundaries. The term may or may not refer to a protein name or to any grammatical class. We call *n-gram *a term that contains *n *words. "The" is thus considered a term, and a one-gram, while "The method" can be called a term, a 2-gram. The *frequency *of a term in a document or set of documents is the number of times the term occurs in the document or set of documents. Here, we identify n-grams in an article by finding all the unique word sequences of length 1 to 5 words that have a frequency greater than one. (We observed that most protein names with high frequency are shorter than 5 words, but this parameter could be varied.)

### Disambiguating biological entity names

**Figure 4:**
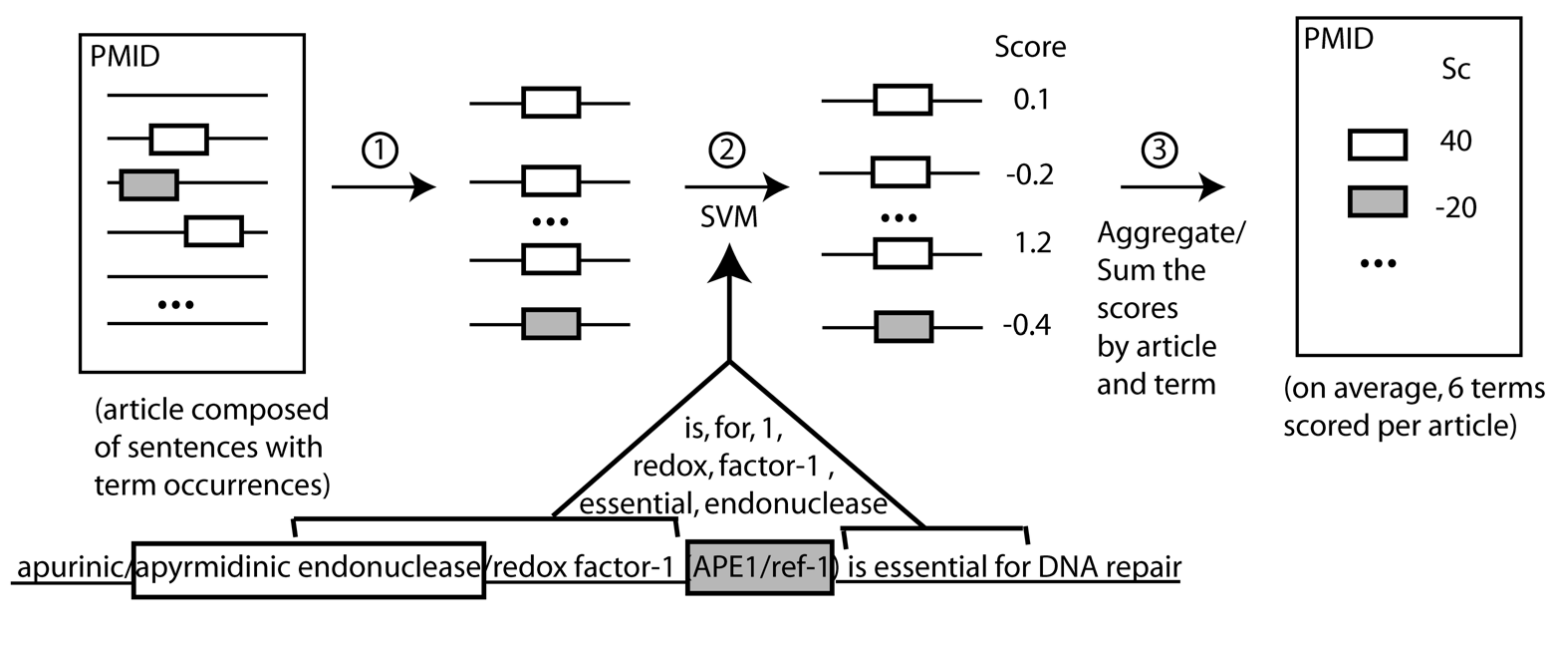
**Steps involved in constructing the catalog of protein references. **Terms are shown enclosed in rectangular boxes. Terms may occur in the context of sentences (when on a horizontal line, left), or in an article (right). Step 1: Articles are split into sentences, and sentences are split into tokens. Tokens roughly correspond to words (see text for details). Tokens with high frequency that are not eliminated by the exclusion lists (see Figure 1) are grouped into n-grams. On the figure, APE1/ref-1 is a n-gram that consists of two tokens: APE1 and ref-1, and can be recognized if the two terms co-occur frequently in sequence in a full length article. When the terms are recognized, each occurrence of a term in sentences of the article is identified. Step 2: Machine learning features are calculated from the context of the term in the article (see text for details) and the support vector machine (SVM) model classifies the context of the term. We obtain the score for each context of a term. In our experimental setup, smaller scores suggest that the context provides little evidence that the term refers to a protein, while larger scores (in absolute values) indicate more support. Step 3: We calculate the combined score (Sc) as the sum of the scores for each occurrence of a given term in a given article. The final catalog consists of a table with one row per term and article. Each row has three columns: PubMedID, term, and Sc.

In an approach similar to [22], we convert terms to features and use a machine learning approach to disambiguate protein names from other names. In contrast to [22], where Hatzivassiloglou et al. used morphology and positional information, we only use the context of the terms in the sentences of the article to make a decision. Furthermore, in our approach, only the terms that are the most frequent in an article are considered for disambiguation. We treat each occurrence of a term in an article separately and then aggregate the predictions to determine one prediction per term and article. This process is illustrated in Figure 4.

### Calculating features for each occurrence of a term in the article

We call *feature *a real-valued number that is used as input to a machine learning algorithm. Let *p(a,t,o) *denote the position of one occurrence *o *of term *t *in article *a*, and *i(w) *be a function that maps each word of the corpus to an integer (a number that uniquely identifies a feature). Such a function could map word "the" to 12 and word "The" to 105, or map both words to the same integer, to wrap cases. The features for term *t *at *p(a,t,o) *are the set of words *W(p(a,t,o)) *that exist in a window of words from *p(a,t,o) *- *l *to *p(a,t,o) *+ *length(t) *+ *l *-1, excluding the term word(s), with *l *the length of the window on either side of the term occurrence and *length(t) *the number of words of term *t*. We define one feature, identified by its index: *i(w) *for each word *w *that occurs in the training corpus. For each word *w *that occurs in this window, and does not belong to the term we set the value of feature *i(w) *to 1. The value of feature *i(w) *is set to zero if the word does not occur within the window. The feature thus gets the same value if the term occurs once or multiple times in the window around a term occurrence. For experiments described in this article, we used a window size of 3.

### Training data set

Our training data set (JBC99) consisted of 1,814 articles (about 520,000 sentences) published in the Journal of Biological Chemistry (JBC) during the last quarter of year 1999. Full length articles were obtained as HTML files and converted to text using the HtmlParser package [25]. Image tags were replaced by their ALT tag, when available, or by whitespace. For most journals that present Greek letters as images, the ALT tag contains a textual representation of the symbol, in these cases, β is replaced by "beta". Presentation tags, such as <p> and <br>, were replaced by whitespace.

### Training SVM models

We trained three support vector machines [26] (also called SVM models). Training SVM models requires training data sets with positive and negative examples. To construct training data sets, we created lists of terms in several categories. To create these lists, we filtered the most frequent terms obtained for each article of the training corpus. We built four lists of non-ambiguous terms: protein/gene names (*PG*), cell names (*C*), process names (*Pr*), and interaction keywords (*IK*). We made sure that the names in these lists were non-ambiguous, that is, that the name, in any sentence context would be a true instance of its class. To construct *PG*, for instance, we included n-grams that match the regular expressions ".+ receptor" or ".+ kinase" (thus, n> = 2), because n-grams that end in "receptor" or "kinase" are very unlikely to be used in a context where they do not refer to proteins. Other terms that could be ambiguous – e.g., SNF, which could be a gene/protein name, or a funding agency (Swiss National Science Foundation) – were not used for training. Regular expressions were used to facilitate the assembly of drafts of these lists, but the lists were carefully inspected and edited manually before training.

Table 4 describes the composition of the training sets built from the non-ambiguous lists described above and indicates the number of terms used for training. Table 5 presents "ξα estimates" after training. The ξα values are conservative estimates of the leave-one-out error that can be computed efficiently after training an SVM [21]. We created three SVM model training sets: *PG*+/*C *-, where *PG *terms are labelled in the positive class, and *C *terms in the negative class. The other sets used for training were *PG*+/*Pr *- and *PG*+/*IK *-, with the same naming conventions. These training set compositions are chosen so that the three SVM models trained from these datasets will give positive scores to terms that are predicted to be in the category PG. Training was performed with the RBF kernel and parameters γ = 0.005 and C = 19.4433 (*PG*+/*C *-), C = 19.0005 (*PG*+/*IK-*), C = 19.1587(*PG*+/*Pr *-).

Binary SVM classifiers assign a predicted class to a term by considering the sign of the output of a trained support vector machine. The output of the SVM is un-calibrated and is not the prior probability of the class given the features [27]. However, the SVM output is correlated with the probability [28] and thus the magnitude of the output can be used as a measure of confidence in the prediction (small absolute values indicate smaller confidence, while larger absolute values indicate stronger confidence).

### Combining predictions for several occurrences of a term in one article

For a SVM model, the individual classification scores for each occurrence of a term in an article were summed, so as to produce three values: Sum_PG+/C-_, Sum_PG+/IK- _and Sum_PG+/Pr-_. We used a simple heuristic expression to combine the three scores into the final Sc score (we summed the three Sum scores and adversely weighted a negative sum in any of the three classifications by multiplying each negative Sum by 50 before adding them). The parameter 50 was chosen empirically to give more weight to negative individual Sum scores. The combined score, *Sc*, is such that greater, positive values have a higher possibility of referring to protein names, and negative values are unlikely to refer to protein names.

### JBC2000 Test set

We assessed the quality of the biological entity name disambiguation with a test set derived from articles published in JBC during the year 2000. The test set is mostly independent from the training set, as the only common points between the articles in the two sets is that they were published in the same journal and formatted with the same conventions.

### Creation of a dictionary of gene and gene product names

Not each term in the protein catalog can be used directly to lookup protein names in articles. A key problem is ambiguity, terms that refer to proteins in certain articles, but refer to other concepts in other articles. In an attempt to reduce ambiguity, we filtered the protein catalog with several heuristics. These heuristics are presented in Figure 3. When applied to the catalog of protein references produced from the "most frequent" terms obtained from 80,528 articles from JBC, EMBO, and PNAS, these filters produce a dictionary of 59,990 terms.

### Evaluation of the dictionary to lookup gene and gene product names in full text

#### Dictionary Test Set construction

We built the NCB14 set with 14 articles selected randomly from articles published in Nature Cell Biology in 2003. Since this journal was not used for the construction of the protein catalog, the style, formatting and names present in these test articles had never been used to develop our method. Furthermore, we did not refine or tune any of the parameters of our method after we started this evaluation. Therefore, the performance values that we report here should be representative of what can be expected when the method is used on new, unseen, but similar full length article materials.

#### Matching names of the dictionary to articles with textractor

Names found in the dictionary were matched to the text of each article in the test sets. When matching n-grams, we match letters and digits and ignore punctuation and special characters except dashes ('-') and dots ('.'). Using this strategy, if A, B and C are words, an "A B-C" in the dictionary will match "A,B-C" and "A#B-C" in the text of an article, but not "A B,C". Using this matching procedure, the name "PKC-zeta lambda" will match "PKC-zeta/lambda" in article PMID 10749857. Matching of words shorter than 6 characters is case-sensitive (i.e., "TnT" will not match "TNT"), while matching of longer words is case insensitive "human topoisomerase II" will match "human TOPOISOMERASE II"). In contrast to other dictionary-based approaches (e.g., [13]), our matching procedure does not allow variations on the words that constitute the name: "receptor" will not match "receptors". Since this matching method is fairly strict, plurals and other variations of protein names must appear explicitly in the dictionary for the name to be matched to the full text.

#### How to define gene/gene product/protein names

The definition of protein that we used to train Textractor differs from the definition used by the human annotators who created the YAPEX corpus [29]. For Textractor, protein names refer to a protein or to the part of a protein, while for YAPEX, proteins must be single entities. The Textractor definition allows for protein domains (e.g., "SH2 domain") and parts of proteins, such as "histone H3 at serine 10", or protein complexes ("the proteasome"). Our definition was chosen pragmatically, since parts of proteins are often mentioned when describing interactions and a long term goal of our project is to extract protein names to support the extraction of information about interactions. We count partial matches as errors when the partial match does not refer to a protein name in itself. For instance, "proteasome" and "the proteasome" are both correct identifications, while "endothelin-converting" is incorrect even if "endothelin-converting enzyme" is a correct match in the same article.

#### Comparing with NLProt

Articles in the HTML format were converted to Unicode text as described under document preprocessing. Both NLProt and the Textractor lookup tool were given the same text material as input. The output produced by NLProt was parsed to extract the protein names recognized by the method. We sorted names to be unique (following the term matching criteria described above), and produced a tab delimited file (first column: PMID; second column: term extracted from this article). This file was merged with the Textractor predictions using the term as unique key. The numbers of occurrences found by each method are also listed. These files were annotated by the authors during the evaluation and are provided in supplementary material to allow comparison with future methods.

#### Evaluation measures

Various performance measures can be used when comparing prediction or extraction methods. Accuracy of a prediction is defined as the percentage of correct predictions, over all the classes of terms predicted. For instance, when predicting protein and non-protein names, accuracy measures how well both protein and non-protein names are predicted. However, accuracy can be misleading when the test set contains one class in a larger proportion than the other. For instance, if the test set contains 10% of terms that are proteins and no proteins names are predicted, the prediction has an accuracy of 90%. For this reason, we prefer the precision and recall measures. The precision is the accuracy measured over one class, for instance measured over proteins. It is calculated as the ratio of correct predictions over the number of predictions made. Recall measures how many elements of one class are predicted in this class. Recall is calculated as the ratio of correct predictions in one class over the total number of instances of this class that could have been predicted. Intuitively, "precision" measures how specific a prediction is when a class is predicted, and "recall" measures how much the prediction method has missed in a class.

To combine and summarize performance values obtained for several articles, we use micro- and macro-evaluation [21,25]. Macro-evaluation averages the values of precision and relative recall calculated in individual articles, while micro-evaluation sums the counts of correct and incorrect predictions over all the articles before calculating global measures.

#### Relative recall

When comparing several prediction methods, we define the *relative recall *of method *i *(among *n *possible methods)as:



where Ci represent the set of all the correct predictions made by method i, Card(set) represents the number of elements in a set, and Union(set1, set2,...) represents the set that is the union of several sets. It can be seen that if any of the methods has perfect recall, the relative recall of each method matches the traditional recall. Furthermore, when the measure is applied with more than two methods, the values of the relative recall will converge towards the recall as the number of methods increases (each method contributes to the union the true positives that is detects, until the union matches the complete set of positives in the data set). The advantage of the relative recall is that only predicted terms need to be evaluated for each prediction method, so that reading the whole article is not required.

Given the observed variability of precision in the articles of our test sets, we assume that the recall of an extraction method will also vary from one article to another. Therefore, it is clear that a measure of recall, however consistent and careful the evaluation, will not be informative if evaluated for a single article. Measuring relative recall lowers the cost of evaluation and makes it possible to compare the recall of several methods over larger article samples.

## Authors' contributions

LS and FC designed, conducted this study, and wrote the manuscript.

## Supplementary Material

Additional File 1 **Full-text lookup tool with dictionary. **This tool implements the lookup approach that is used for evaluation of the protein dictionary in the manuscript. The Java Archive (Zip format) contains a copy of the protein dictionary that is also available from our web site. (Additional instructions will be printed to the console.) Click here for file

## Figures and Tables

**Table 1:** Example of terms that occur most frequently in an article (selected from article with PMID: 10506131).

**Term**	**Frequency**	**Category**
GnRH	79	a
side chain	60	a
the GnRH receptor	30	a
d7.49 318	38	a
the	503	b
of	334	b
and	212	b
expression	49	b
*d7.49*	*38*	*a*
*the GnRH*	*30*	*a*

Terms in italic are not considered for further analysis because their frequency in the document is the same as the frequency of a longer term that exactly contains them ("d7.49" is contained in "d7.49 318" and their frequencies are the same).

This indicates that the shorter term never occurs alone in the document.

**Table 2:** Evaluation results for the NCB14 dataset.

	**Textractor**				**NLProt**				**Union**
**PubMedID**	**Correct**	**Incorrect**	**Precision**	**Rel. Recall**	**Correct**	**Incorrect**	**Precision**	**Rel. Recall**	**Correct**

12629548	41	19	68%	67%	37	77	32%	59%	63
12629549	38	3	93%	84%	32	79	29%	40%	50
12640462	23	17	58%	100%	1	13	7%	4%	23
12640463	18	17	51%	36%	46	77	37%	87%	53
12640464	67	18	79%	72%	50	26	66%	50%	100
12669071	14	8	64%	54%	19	35	35%	73%	26
12669072	14	3	82%	65%	18	27	40%	51%	26
12669073	27	1	96%	81%	25	31	45%	53%	39
12669075	19	7	73%	73%	16	26	38%	47%	26
12669077	19	5	79%	73%	16	38	30%	39%	28
12669082	86	26	77%	68%	82	87	49%	65%	126
12669083	64	13	83%	58%	81	74	52%	72%	112
12679784	25	7	78%	58%	26	37	41%	60%	43
12692559	54	26	68%	60%	67	77	47%	55%	99

**micro-evaluation**	509	170	**75%**	**63%**	516	704	**42%**	**63%**	814
**macro-evaluation**			**75%**	**67%**			**39%**	**54%**	

**Table 3:** Evaluation results for the PMC15 data set.

**Evaluation**	**Performance Measure**	**YAGI**	**Textractor**	**NLProt**
micro-evaluation	precision	43%	66%	34%
micro-evaluation	rel. recall	63%	31%	41%
micro-evaluation	rel F-1	51%	42%	37%
macro-evaluation	precision	40%	53%	29%
macro-evaluation	rel. recall	66%	30%	37%
macro-evaluation	rel F-1	52%	40%	34%

**Table 4:** Composition of the training sets

**Training lists**	**PG**	**C**	**IK**	**Pr**
**# n-grams, n>=2**	304	193	111	254
**# occurrences in articles where the n-gram is most frequent**	16,543	10,862	5,853	12,547

**Table 5:** Performance estimators after training.

**Training sets**		PG+/C-	PG+/IK-	PG+/Pr-
**ξα estimates**	recall >=	73.96%	78.62%	58.48%
	precision >=	72.82%	75.52%	57.75%
	error <=	32.37%	34.61%	47.93%
